# Optimization of Wind Driven RO Plant for Brackish Water Desalination during Wind Speed Fluctuation with and without Battery

**DOI:** 10.3390/membranes11020077

**Published:** 2021-01-20

**Authors:** Emad Ali, Mourad Bumazza, Ali Eltamaly, Sarwono Mulyono, Muath Yasin

**Affiliations:** 1 Chemical Engineering Department, King Saud University, Riyadh 11421, Saudi Arabia; mouradb@ksu.edu.sa (M.B.); sarmulyoprayitno@ksu.edu.sa (S.M.); 439105965@student.ksu.edu.sa (M.Y.); 2 Sustainable Energy Technologies Center, King Saud University, Riyadh 11421, Saudi Arabia; eltamaly@ksu.edu.sa; 3 Electrical Engineering Department, Mansoura University, Mansoura 35516, Egypt

**Keywords:** membrane desalination, reverse osmosis, wind power, optimization, wind intermittency

## Abstract

This work aimed to carry out an optimal investigation of the design and operation of a large capacity reverse osmosis (RO) desalination plant powered by wind energy. Different scenarios involving two design options, such as using storage tanks or batteries, and operation options, such as using variable or fixed feed pressure, were analyzed and optimized. In addition, another operation option, of using a fixed number of RO vessels or a varying number of active RO vessels, was also considered. It was found that an optimized plant using storage tanks can provide a less expensive water cost and a less complicated plant structure. Moreover, the use of a variable feed pressure can help in attenuating the disturbances incurred in the form of wind intermittency. Conversely, the use of fixed feed pressure and constantly supplied power per vessel can run the RO units smoothly, leading to a predictable production rate. However, this requires operating the plant on different active sets of vessels each hour, which mandates additional automatic control systems. The water cost when storage tanks are utilized can be as low as 7.42 $/m^3^, while it is around 19.7 $/m^3^ when a battery is used.

## 1. Introduction

Water is an inevitable element of human existence. Besides drinking, humans use water for every aspect of life. A continuous supply of potable water is becoming extremely challenging due to rapidly growing population, urbanization, and industrialization around the globe. For decades, the desalination of brackish water [1] and seawater [2] has been the main method of providing fresh water to regions suffering from water resource scarcity. However, as energy demand and cost keep increasing, and as fossil fuels are creating environmental harm, conventional desalination technology is facing true challenges. Reverse osmosis (RO) comprises 65% of the globally installed desalination technologies [3], and is employed in diverse industrial applications [4]. RO stands as the least energy demanding desalination technology. According to Dashtpour and Al-zubaidy [5] RO consumes 3 to 10 kW to produce one cubic meter of freshwater from seawater. A large fraction of the energy consumption is associated with pressurizing the feed water. The cost of electric pumping power varies with fluctuating fossil fuel prices. Charcosset [6] reported an 11% variation in the specific water cost for a 25% variation in energy cost. In order to overcome the fluctuating cost of energy, and its detrimental effect on the environment, it is judicious to utilize renewable energy sources to power desalination plants. In this regard, combining RO desalination systems with solar or wind energy is a promising alternative that is the subject of tremendous research activities.

Saudi Arabia is a vast and arid country that has a growing demand for freshwater and electricity. The country’s 2030 vision aims to increase the use of renewable energy sources with the goal of generating 40 GW of solar energy and 17 GW of wind energy [7]. These renewable sources of energy will notably serve as sources of electricity and to power autonomous, decentralized, and off-grid water desalination and treatment systems in the remote areas of the country.

While the desert climate of the country is known to provide it with an excellent potential source of solar energy, a recent survey showed that wind energy is also a viable source in the country, with many areas having a wind speed of over 3.5 m/s [7]. It becomes imperative, therefore, to study the potential of harnessing the available wind energy in these areas to produce potable water from local aquifers via autonomous and decentralized RO desalination plants. Wind energy for RO systems also has the potential to be environmentally friendly and cheaper compared to the other fuel-based energy sources [8].

The use of wind to power RO systems has been studied extensively in the literature [9,10]. Efforts have been focused on addressing the reliability of such systems in the face of wind intermittencies, which represents one of the main weaknesses of wind/RO systems. Park et al. [11], for instance, studied a wind-powered RO system without energy storage and concluded that reliable control strategies are needed to tackle intermittent operations, especially for high salt feed concentrations. Carta et al. [12] investigated experimentally the use of stand-alone operation of off-grid wind farms to supply energy to various desalination plants including RO systems. The authors concluded positively on the technical feasibility of such systems, provided they operate with a variable capacity. Charrouf et al. [13] showed that artificial neural networks can be successfully used to achieve the smooth power management of a reverse osmosis unit fed by hybrid renewable energy sources, solar panels, wind turbines, and battery banks. Peng et al. [14] investigated the use of different evolutionary algorithms to determine the optimum size under wind intermittency of a hybrid renewable energy system comprised of a wind turbine, a photovoltaic panel, a battery bank, and reverse osmosis desalination. Cabrera et al. [15] investigated the use of different machine learning approaches for the analysis of the performance of a seawater RO system powered by intermittent wind energy. The system was analyzed under fixed and variable feed pressure and flow rates. Carta et al. [16] investigated the use of a small-scale prototype (Seawater RO) SWRO driven by wind energy. The system was designed to continuously adapt its energy consumption to the variable power supplied by a wind turbine by the use of a supercapacitor bank. Richards et al. [17] also studied the performance of a desalination plant aided by a supercapacitor energy bank under real wind fluctuations. Lai et al. [8] discussed the different strategies for attenuating the detrimental effects of fluctuating and discontinuous wind energy on the operation of RO desalination plants. The proposed solutions consisted of using energy storage, integrating different types of renewable energy sources, and modifying the system operating conditions. Mohamed and Papadakis [18] studied the economics of using hybrid solar and wind energy aided by a battery bank for RO water desalination systems. They pointed out that using pressure energy, recovery devices can reduce energy consumption by 48%, which in turn improves the overall economics. Khiari et al. [19] investigated the use of isolated hybrid PV-wind systems to purify brackish water without batteries. They highlighted the importance of using field-oriented control to stabilize the electricity in the power line. Moreover, they underscored the use of energy management systems for the proper sharing of energy sources. Additionally, recent review papers on the combination of solar/wind sources with RO technology can be found in [20,21]. Basically, designing and operating a wind driven RO plant is a challenging and complicated task, due to intermittent and fluctuating wind speed. Hybrid systems are also more complicated to design and operate because they require additional instrumentation and control components [19]. In addition, hybrid systems are costly, since photovoltaic systems have a higher capital cost [18].

In general, designing an RO plant that provides 100% satisfaction of the municipal water demand on an hourly basis, and to some extent yearly, would require a large number of turbines, vessels, and batteries, which increase the water cost. Hence our goal here is to investigate whether adapting the operating conditions, such as feed pressure, delivered power per vessel, or several active vessels, can help to reduce the reliance on large quantities of turbines and batteries, and consequently reducing the water production cost. In this work, we present a techno-economic analysis of an RO plant that supplies 100% of the water demand for a specific site in Saudi Arabia by desalinating brackish water. Specifically, different design and operation scenarios for the RO plant powered by wind energy, with and without batteries, will be simulated. The scenarios differ on the selected production scheme, i.e., either annually or hourly, and differ on the structure of the plant, i.e., fixed or variable pressure and/or a number of vessels. In both cases, the design parameters, such as the number of wind turbines, number of vessels, number of batteries, and the set point of feed pressure will be optimized to meet certain criteria, such as minimizing water cost, loss of hourly production, or loss of annual production. This will be carried out via hourly simulation and economic analysis on a yearly basis. The diverse scenarios will be compared, and the related characteristics of these structures will be highlighted. Hence, this analysis will help to choose the best design scenario, in terms of the lowest water cost, that fits given constraints and plans.

## 2. The Wind Driven RO Plant

**Figure 1:**
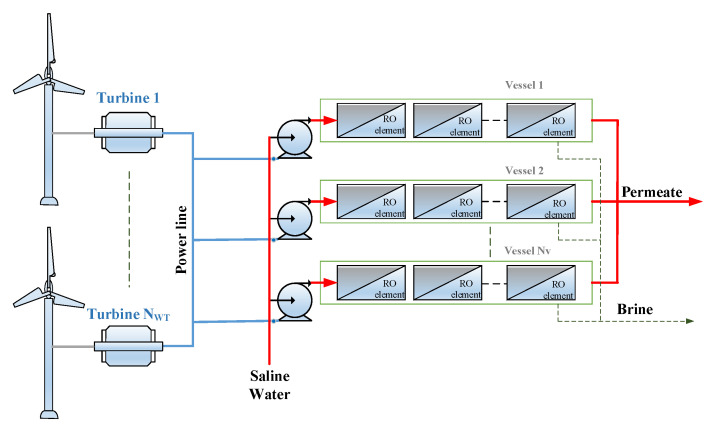
Wind-driven reverse osmosis (RO) plant structure.

**Figure 2:**
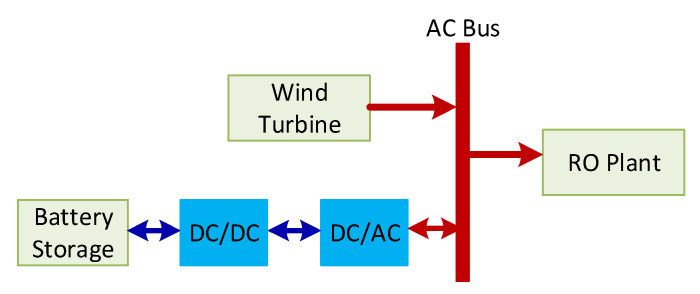
AC coupled configuration for the power line.

The structure of the RO plant powered by wind energy to provide the selected site with the daily water demand is depicted in Figure 1. This desalination plant structure should provide 2592 m^3^/h of freshwater over the entire year to satisfy the required demand of Arar province in Saudi Arabia [22]. The required power load to meet such production was estimated previously [22] to be around 1780 kW every operating hour. This configuration comprises a network of RO modules, water pumps, and wind turbines. The RO network consists of several parallel RO vessels, each of which comprises eight RO elements with three leaves [23,24]. Practically, the maximum feed flow rate to a single RO module is limited by upper and lower bounds of 15 and 2.5 m^3^/h, respectively for safe operation [25]. Consequently, and because of other constraints, a single RO vessel cannot supply the needed water demand of the site. Therefore, a minimum number of RO vessels (*N_v_*) must be used to sustain the expected production. In this case, *N_v_* equals the hourly water demand divided by the permeate production of a single vessel, provided that all vessels are identical and operating at the same input conditions. The total power load of the RO plant equals *N_v_* multiplied by the necessary pump power per vessel. Considering the variations in wind speed and the capacity of the wind turbine, a single wind turbine may not provide the needed power load. Thereby, a number of wind turbines are required as shown in Figure 1. Due to the intermittency of the wind speed, the generated power may reach zero, even if a large number of turbines are implemented. Thereby, the common practice is to incorporate energy storage devices such as battery banks to compensate for the power losses. The topology of the power system components is illustrated in Figure 2. Considering a wind turbine with a regulated AC output voltage, its generated power can be connected directly to the AC-bus. The battery is linked to the AC bus through bidirectional DC/DC and DC/AC converters. When the generated wind power exceeds the RO load, the surplus power is used to charge the battery. Conversely, if the generated power is less than the load, energy is withdrawn from the battery to compensate for the gap. The selection of the total number of vessels, total number of wind turbines, total number of batteries (or equivalently total storage capacity), and the operating condition for each vessel will be optimized. The optimization objective will be based on certain design criteria, as will be discussed in the following sections.

## 3. Plant Design Components Description

### 3.1. The Wind Power System

**Figure 3:**
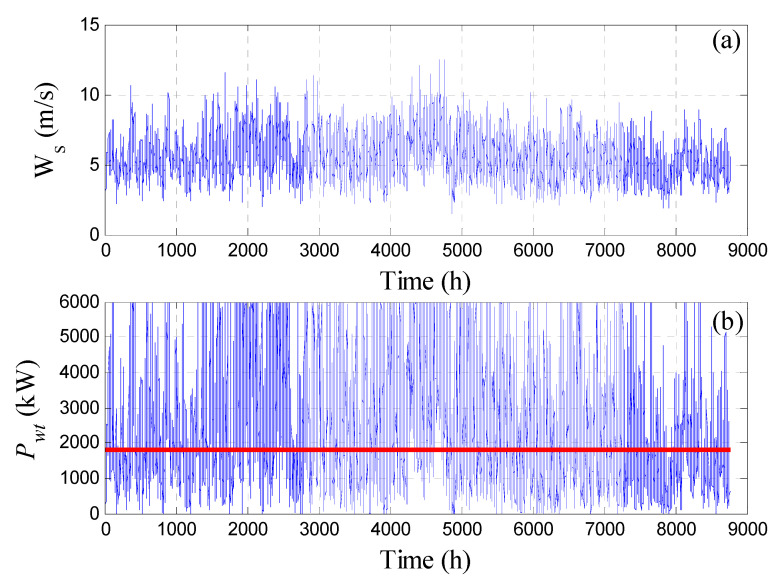
Annual wind property profile for the site: (**a**) wind speed; (**b**) generated power; constant line denotes the target value; solid line: required power load.

In this study, we choose AE-Italia [26] data for simulating the wind turbine. This chosen wind turbine has a rated power of 60 kW at a rated wind speed of 8 m/s, a cut-in wind speed of 2.5 m/s, and a cut-out wind speed of 25 m/s. It has a scale parameter of 5.5661, a shape parameter of 2.5649, and a capacity factor of 0.3325. Based on the rated power and capacity factor, ninety AE turbines are needed to provide an average power of 1780 kW, which is the load required by the RO plant to produce the targeted water demand. The hourly generated power (*P_w_*) from a single wind turbine is given by [27]:

(1) $$P_{w}(u)=\left\{\begin{array}{ccc} 0 & & u\leq u_{c}\&u\geq u_{F}\\ P_{r}\frac{u^{k}-u_{c}^{k}}{u_{r}^{k}-u_{c}^{k}} & & u_{c}\leq u\leq u_{r}\\ P_{r} & & u_{r}\leq u\leq u_{F} \end{array}\right\}$$

where *P_r_* is the wind turbine rated power, *u* is the wind speed, and *u_c_*, *u_r_*, and *u_F_* are the wind turbine cut-in, rated, and cut-off speeds, respectively. Hence, the average power per turbine, the total generated power from all turbines, and the supplied power per vessel are simply:

(2) $$P_{w,av}=C_{F}P_{w}$$

(3) $$P_{wt}=P_{w}N_{WT}$$

(4) $$P_{wv}=P_{wt}/N_{v}$$

where *C_F_* is the capacity factor, *N_WT_* is the total number of turbines, and *N_v_* is the total number of vessels. The available hourly wind speed for the selected site and the corresponding generated power per turbine are depicted in Figure 3. The figure shows clear wind fluctuation and intermittency, as well as the generated power during the year. This makes producing a consistent water flow rate a very difficult task. Since the available wind speed data is on an hourly basis, the design procedure in this work will also be based on an hourly time frame.

### 3.2. The RO Model

The modeling equations that describe the water separation in a typical RO were developed previously [28]. The typical RO model is well established in the literature; therefore, the governing model equations are briefly described in the following:

For each RO vessel, the feed flow rate (*Q_fv_*) can be obtained from the following relation [29]:

(5) $$Q_{fv}=\frac{3600\times P_{wv}}{P_{f}\times 1\times 10^{5}\eta_{p}^{-1}}$$

where *P_wv_* is the power supplied to the feed pump, also defined as the available power per vessel, *η_p_* is the pump efficiency, and *P_f_* is the feed pressure. Knowing the recovery ratio (*R_c_*), the permeate production per vessel (*Q_pv_*) is evaluated as follows:

(6) $$Q_{pv}=Q_{fv}R_{c}$$

The mass balance around the RO unit for water and salt gives:

(7) $$Q_{fv}=Q_{pv}+Q_{cv}$$

(8) $$Q_{fv}C_{f}=Q_{pv}C_{p}+Q_{cv}C_{c}$$

where *Q_cv_* is the brine volumetric flow rate, *C_f_*, *C_p_,* and *C_c_* are the salt concentrations in feed, permeate, and brine, respectively. The bulk flow rate (*Q_b_*) and salinity (*C_b_*) are taken as the average values:

(9) $$Q_{b}=\frac{Q_{fv}+Q_{cv}}{2}$$

(10) $$C_{b}=\frac{C_{f}+C_{c}}{2}$$

The water flux (*J_w_*) is considered to be proportional to the pressure difference as follow [30]:

(11) $$J_{w}=A\left(\Delta P-\Delta \pi \right)$$

where the transmembrane pressure drop ΔP [30] is defined as follows:

(12) $$\Delta P=P_{f}-P_{b}-P_{drop/2}$$

(13) $$\Delta \pi =b_{\pi }\left(C_{m}-C_{p}\right)$$

π is the osmotic pressure, *P_f_* and *P_b_* are feed and brine pressure, *P_drop_* the pressure drop, *C_m_* the average salt concentration at membrane wall, and b_π_ is the osmotic coefficient, which is given as follows:

(14) $$b_{\pi }=\pi /C_{b}$$

The osmotic pressure, *π*, is estimated from the salt concentration as follows [29]:

(15) $$\pi =1.12T\sum {\overset{¯}{m}}_{i}$$

where $\sum {\overset{¯}{m}}_{i}$ is the sum of all molality’s of dissolved ions (ppm) and *T* is the bulk temperature.

The pressure drop along the membrane length (*P_drop_*) can be approximated using the following expression [30]:

(16) $$P_{drop}=9.5\times 10^{8}{\left(\frac{Q_{f}+Q_{c}}{2\times 3600}\right)}^{1.7}$$

Concentration polarization is common for RO processes, thereby the water flux (*J_w_*) and the permeate salinity (*C_p_*) can be related to the salt concentration at the membrane surface as follows [31,32]:

(17) $$J_{w}=A\left[\Delta P-b_{\pi }\left(\frac{BC_{b}\exp\left(J_{w}/k_{s}\right)}{J_{w}+B\exp\left(J_{w}/k_{s}\right)}\right)\exp\left(J_{w}/k_{s}\right)\right]$$

and

(18) $$C_{p}=\frac{BC_{b}}{B+J_{w}\exp\left(\frac{J_{w}}{k_{s}}\right)}$$

(19) $$C_{m}=C_{p}+\left(C_{b}-C_{p}\right)e^{J_{w}/k_{s}}$$

where *k_s_* is the mass transfer coefficient and *B* is the membrane solute permeability.

The nonlinear algebraic Equations (5)–(19) can be solved iteratively, as described in Appendix A, to evaluate the permeate concentration (*C_p_*), the overall production rate (*Q_w_*), and the total production of the plant:

(20) $$Q_{w}=J_{w}A_{s}n_{e}n_{l}$$

(21) $$Q_{wt}=Q_{w}N_{v}$$

The RO recovery ratio is defined as follows:

(22) $$R_{c}=\frac{Q_{w}}{Q_{fv}}$$

The mass transfer coefficient (*k*_s_), used in Equations (17)–(19), can be estimated using the following correlations [33]:

(23) $$Sh=0.065Re^{0.865}Sc^{0.25}$$

where:

(24) $$Sh=\frac{k_{s}}{D_{AB}};Re=\frac{d_{h}u}{µ};Sc=\frac{v}{D_{AB}}$$

where *Re*, *Sc,* and *Sh* are the Reynolds, Schmidt, and Sherwood numbers, respectively.

The velocity in the feed channel (*u*) that contains a baffle is given by:

(25) $$u=\frac{Q_{b}}{wh_{sp}\varepsilon }$$

where *d_h_*, *h_sp_*, and *ε* are the baffle parameters, and *w* is the width of the membrane. *D_AB_* is the diffusivity coefficient. The kinematic viscosity (*ν*) for brackish water can be calculated through the following correlation [34]:

(26) $$v=0.0032+3.0\times 10^{-6}C_{b}+4.0\times 10^{-9}C_{b}^{2}$$

The value of diffusivity (*D_AB_*) is given as 5.5 × 10^−6^ m^2^/h [29].

The RO model equations are solved via two algorithms based on the feed pressure status. The feed pressure can be either variable within the RO model, or specified externally, as described by the method of solution in Appendix A. Table 1 shows the RO membrane specifications [35] used in this paper. These specifications are for a spiral wound module, and include hydraulic diameter, the specific surface area of the spacer, the void fraction, and the channel height.

### 3.3. Water Storage Mechanism

When dealing with the annual production demand, water storage tanks are required. In this case, the surplus of water produced each hour beyond the hourly demand is transferred to storage tanks for later use. Whenever the hourly produced water is less than the hourly demand, the supplied water is compensated by withdrawing the deficit from the storage tanks. Therefore, the hourly stored capacity (*TS*) can be computed for any sampling hour (*t_k_*) as follows:

(27) $$TS\left(t_{k}\right)=TS\left(t_{k=1}\right)+Q_{w}\left(t_{k}\right)-Q_{P}\left(t_{k}\right)$$

Subject to the following constraint:

(28) $$TS\left(t_{k}\right)>0$$

The above is applied for *k* = 1 to 87,600 h, comprising one year of operation. Note that no upper limit on the storage capacity is imposed because no excess permeate shall be rejected.

To assess whether the plant water production meets the annual requirement, the loss of production probability is used as a criterion. We define the loss of production (*LPR*) as:

(29) $$LPR\left(t_{k}\right)=Q_{P}\left(t_{k}\right)-Q_{w}\left(t_{k}\right)$$

Hence the annual loss of production probability (*LPRA*) is:

(30) $$LPRA=\frac{\sum_{k=1}^{N}LPR\left(t_{k}\right)}{\sum_{k=1}^{N}Q_{P}\left(t_{k}\right)}$$

where *N* is 8760 in this study. A value of 1 for *LPRA* denotes full loss of production, while a value of 0 means the required production is fully satisfied. Note that *LPRA* can have a negative value which indicates a surplus of water beyond the targeted demand. A negative *LPR* also requires a larger tank capacity to store the abundance of water, which increases the capital cost, but at the same time reduces the specific water cost. It should be noted that *LPRA* is used with the tank storage capacity when the design criteria are based on meeting the annual production rate. Note also that the number of required storage tanks (*N_T_*) can be calculated directly by dividing the storage capacity by the size of a single tank, which is 30 m^3^ in this study. Since *N_T_* and *TS* are directly related, we will use both terms interchangeably throughout the text. Although the design procedure will be based on minimizing *LPRA*, the hourly water balance will be computed for assessment. The hourly production will be distributed according to Equation (27). This means that at each hour, when water is produced in excess, the excess will be stored in the tanks, while when a shortage of water is produced, water is withdrawn from the tanks and added to the delivery line to compensate for the losses. Thereby, the hourly production loss in the pipelines is defined as follows:

(31) $$LPRb\left(t_{k}\right)=LPR\left(t_{k}\right)-\left(TS\left(t_{k-1}\right)-TS\left(t_{k}\right)\right)$$

(32) $$LPRHb=\frac{\sum_{k=1}^{N}\left|LPRb\left(t_{k}\right)\right|}{\sum_{k=1}^{N}Q_{P}\left(t_{k}\right)}$$

### 3.4. Energy Storage Mechanism

If the plant strategy is to supply the exact hourly water demand, then the hourly loss of production probability is used to assess the design criterion. The hourly loss of production probability is defined as follows:

(33) $$LPRH=\frac{\sum_{k=1}^{N}\left|LPR\left(t_{k}\right)\right|}{\sum_{k=1}^{N}Q_{P}\left(t_{k}\right)}$$

As before, a value of 1 for *LPRH* denotes full loss of production, while a value of 0 means the required hourly production is fully satisfied. Since the wind power is intermittent, the generated wind power may not be enough to provide the required load, and hence the required hourly production rate. In due course, a battery bank must be used to compensate for the loss of power. The common practice in such cases is that the battery bank is charged when a surplus of power is variable, and discharged whenever a loss of power prevails. This procedure is expressed as follows.

During the charging phase, the battery storage at any instant (*t_k_*) is given by [36]:

(34) $$Sb\left(t_{k}\right)=Sb\left(t_{k-1}\right)+\left(P_{w}\left(t_{k}\right)-\frac{P_{L}\left(t_{k}\right)}{\eta_{inv}}\right)\eta_{bat}$$

where *P_L_* is the required power load and *η_inv_* is the converter/inverter efficiency, taken to be 0.95 in this study [36].

While during the discharging phase, the battery storage capacity is given as follows:

(35) $$Sb\left(t_{k}\right)=Sb\left(t_{k-1}\right)-\left(\frac{P_{L}\left(t_{k}\right)}{\eta_{inv}}-P_{w}\left(t_{k}\right)\right)\eta_{bat}$$

Subject to:

(36) $$0<Sb\left(t_{k}\right)\leq Sb^{max}$$

where *P_w_* is the generated wind power per turbine and *η_bat_* is the battery efficiency, set to 1 during discharging and 0.85 during charging [36]. The battery storage capacity is limited by an upper value (*Sb*^max^). This upper limit will be optimized to minimize the water cost. Note that when storing the power as energy we consider the power to be numerically equal to the energy within one hour of operation. The concept of battery storage is based on the fact that the generated power fluctuates below and above the required load. Therefore, to assess the loss or excess of generated power, the loss of power supply will be used [36]:

(37) $$LPS\left(t_{k}\right)=P_{L}-P_{wt}\left(t_{k}\right)$$

where *P_wt_* is the total generated power. The corresponding loss of power probability is defined as follows:

(38) $$LPSP=\frac{\sum_{k=1}^{N}LPS\left(t_{k}\right)}{\sum_{k=1}^{N}P_{L}}$$

As mentioned earlier, a value of 1 means the required load is completely unsatisfied, a value of 0 means the required load is fully satisfied, and a negative value denotes that a surplus of power is generated beyond the demand. A negative value for *LPSP* also requires larger battery storage capacity, which directly increases the capital cost. The expression for *LPS* (Equation (37)) evaluates the loss of power without using batteries. When using batteries, *LPS* is modified as follows [37]:

(39) $$LPSb\left(t_{k}\right)=P_{L}-\left(P_{wt}\left(t_{k}\right)+Sb\left(t_{k-1}\right)-Sb\left(t_{k}\right)\right)\eta_{inv}$$

The loss of power probability will still be estimated using Equation (38) but using *LPSb* instead of *LPS*.

### 3.5. Water Cost Estimation

The water production cost is an important criterion for designing the wind-driven desalination system. Using the common procedure for estimating the fixed investment and operating cost [30,38], the details of the cost components, as well as the related correlations, are listed in Table A1 and Table A2 in Appendix B. In addition, the values of the cost parameters are given in Table A3. Taking the total annual cost to be the sum of the annual operating cost and annualized capital cost, the specific water cost (*Wc*) can be computed as follows [39]:

(40) $$Wc=\frac{TOC+ACC}{Q_{wr}\times w_{hy}}$$

where *TOC* is the total annual operating cost, *ACC* the annualized capital cost, and *W_hy_* the annual operating hours. It should be noted here that for simplification the cost of water storage tanks is based on the storage capacity instead of the number of tanks. The capital cost of storage tanks is included in the water cost analysis, but their capacity or number is not optimized. This is because storage tanks are essential components of the desalination plant, as it is common to store water for further treatment before deploying it to the municipal facilities. Moreover, no limit should be enforced on the maximum capacity, because no pure water should be rejected. On the other hand, the cost of batteries and inverters are included in both the capital investment and operating costs. In both cases, the cost is related to the battery capacity instead of the number of units, to simplify the calculations. The number of required batteries can be easily estimated by dividing the total required capacity by the capacity of a single battery. Here we choose the battery to be Surrettee-6CS25P models (6 V, 1156 Ah, 9645 kW h) [27,40], in order to calculate the needed number of units. The maximum capacity of the total energy storage is optimized in this study when the hourly production criterion is chosen. Note that optimizing the battery capacity is equivalent to optimizing the number of batteries. The battery storage capacity is preferred because it is a real variable, while the number of batteries is an integer variable. Note also that we will use the term storage capacity (*Sb*) and the number of batteries (*N_b_*) interchangeably, since they are directly related.

## 4. Design Formulation, Procedure, and Scenarios

**Figure 4:**
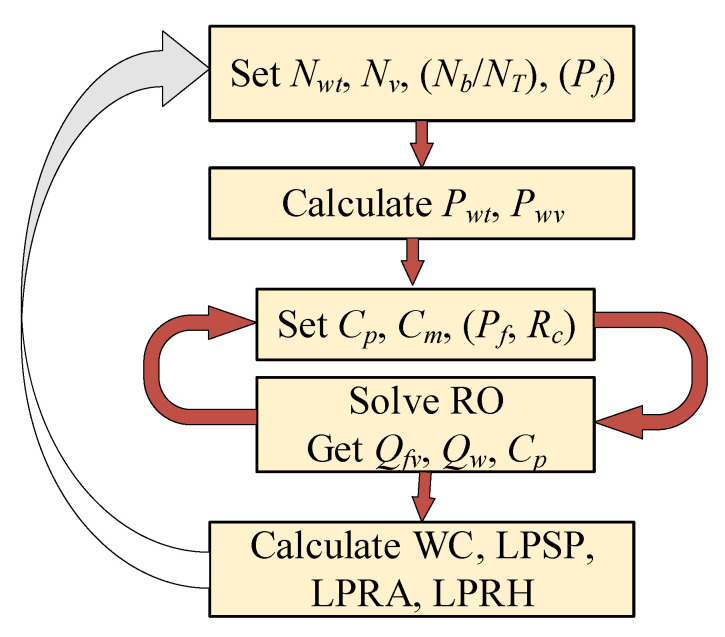
Overall design procedure organigram.

The organigram in Figure 4 shows the overall design procedure. It describes the main route of the design calculations, whereas the other details are given elsewhere. For example, the solution of the RO model is given in Appendix A, while the calculation of the water cost (*WC*) is given in Appendix B. As the organigram illustrates, the design procedure consists of two loops. The outer loop (design loop) fixes the number of vessels, number of turbines, and number of batteries (*N_v_, N_WT_, N_b_*), and in some cases *P_f_*, to satisfy the design criteria, i.e., water cost, loss of hourly production, or loss of annual production. The description and formulation of the design criteria, such as *LPSP*, *LPRH*, and *LPRA*, were discussed in the previous sections. The outer loop specifies and supplies the total generated power (*P_w_*) and the available power per vessel (*P_wv_*) to the inner loop. Given *P_wv_*, the inner loop (RO loop) solves the RO model to estimate *P_f_*, *Q_f_*, and *C_p_*. The latter are used to estimate the design criteria, *WC*, *LPSP*, *LPRH*, and *LPRA*, necessary for the outer loop iteration. The inner loop is also solved iteratively, as described in Appendix A. Note that *P_f_* is placed between brackets to highlight its options. For example, *P_f_* is specified in the inner loop when a variable pressure operation option is selected, while it is specified in the outer loop when the fixed pressure operation option is chosen. Furthermore, recovery ratio (*R_c_*) is set as a design variable for variable pressure mode, and a free variable for constant pressure mode. Similarly, the inclusion of *N_b_* or *N_T_* depends on the design option. For the hourly production option, only *N_b_* is involved, while for the annual production option, only *N_T_* is considered.

The wind powered RO plant has several overlapping design parameters, such as the supplied power per vessel, RO feed pressure, number of RO vessels to be operated simultaneously, number of water storage tanks, and number of energy storage systems. Therefore, several scenarios for the design and operation of the overall plant exist. The best scenario should meet the production target at the minimum cost.

**Figure 5:**
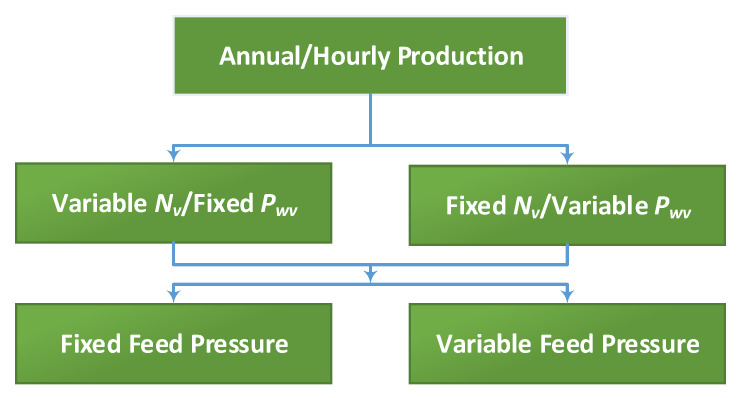
Different design scenarios based on different options for the design variables.

Figure 5 demonstrates the possible scenarios associated with the above design procedure. The RO plant can be designed for annual production, or hourly production, modes. In the annual mode, the goal is that the sum of the hourly production over a year meets an annual target. In the hourly mode, the objective is to meet the specific hourly production target. The annual mode is simpler and less expensive, as it requires only regular tanks for water storage. However, it needs water supply scheduling and may suffer from a shortage in the water supply on some occasions. The hourly mode is more expensive and complicated because it uses energy storage systems to compensate for the power supply loss during low wind speed periods.

The hourly production approach necessitates the use of batteries to store the hourly excess energy for later use during periods of loss of wind power. This is because the exact power load must be provided each hour to obtain the desired hourly production. This incurs extra capital and operating costs, not only because of the battery itself, but also because of the additional supplementary instrumentation and accessories, such as inverters/converters. Furthermore, some generated power will be lost because of the inherent efficiency of these devices, as well as the self-discharge of the batteries. Moreover, the operation becomes more complicated due to the use of automation for charging and discharging the batteries.

Both production approaches will be examined and compared under different scenarios. These scenarios are based on different options for the overlapping design parameters, *N_v_*, *P_wv_*, and *P_f_*. A fixed number of RO vessels (*N_v_*) over the entire year will be tested as the independent variable (design parameter). In this case, the supplied power per vessel (*P_wv_*) will vary with generated wind power (*P_w_*), as the *P_wv_* is simply *P_w_* divided by the fixed number of vessels. Note that *P_wv_* will be distributed evenly over the entire RO vessels. Of course, *N_v_* will be the design parameter to be optimized numerically along with other design parameters to obtain the desired production scheme. Another scenario is to use a fixed *P_wv_* as an independent variable, while *N_v_* is allowed to vary with wind speed. Usually, *N_v_* cannot be changed during design calculation, but it can be during operation by using automatic control to disable and enable a selected number of vessels for certain periods. In this study, we consider this case for comparison purposes and to assess its benefits. In this design scenario, *P_wv_* will be fixed over the entire year. Alternatively, it can be independently varied each hour, however, this will increase the number of design variables to more than 8760, which will make the simulation computationally intensive and intractable. Hence, this option will not be considered.

Other options are based on using variable or fixed feed pressure. To clarify this notion, we define the feed pressure as a variable when determined by the inner loop (RO loop), and fixed when determined by the outer loop. Hourly variable feed pressure is commonly used to solve the RO model numerically, as described in Appendix A. In due course, *P_f_* is optimized at each hour of operation to make the RO process achieve a predefined recovery ratio and permeate quality. Hence, *P_f_* is not used as a design parameter in the design loop. Alternatively, feed pressure can be incorporated in the design phase and imposed as a fixed value in the inner loop (RO model). Specifically, *P_f_* will be fixed over the entire year. In fact, the design loop can still use fixed *P_f_* within an hour, but it differs at each hour of the year. However, like the hourly independent variable *P_wv_*, this situation will be computationally intensive. Conceptually this option is similar to the variable feed pressure mode, with the exception that the former is specified by the design loop, while the latter by the RO loop. Nevertheless, this approach will be dealt with when considering a short window of the annual operation, i.e., one day or a week. The idea is to reduce the number of decision variables, to make the numerical optimization tractable. It should be noted that, in reality, a design procedure that considers independently hourly variable *P_wv_*, *N_v_*, or *P_f_* requires prior knowledge of the wind speed variations over the entire year. Therefore, such cases should be implemented during real time operation using feedback control systems. Nevertheless, the design procedure shown in Figure 4 is achieved for any selected scenario via solving the following optimization problem:

(41) $$\underset{N_{WT},\;N_{v},\left(Sb^{\max},\;P_{f}\right)}{\min}\varphi =WC,\;LPRH,\;LPRA$$

Subject to

(42) $$20\leq N_{WT}\leq 100$$

$$400\leq N_{v}\leq 1200$$

(43) $$1\times 10^{6}\leq Sb^{max}\leq 5\times 10^{9}$$

$$3\leq P_{f}\leq 40$$

$$f\left(P_{f},C_{p},C_{m}\right)=0$$

$$C_{ineq}\leq 0$$

$$C_{eq}=0$$

Since we have different scenarios, the cost index (objective function) can include any of the criteria, *WC*, *LPRH*, or *LPRA*. The optimization problem may contain nonlinear equality or inequality constraints (*C_eq_*, *C_ineq_*). The components of these nonlinear constraints will be revealed in the discussion section. The number of wind turbines and vessels are the default design parameters, i.e., included in all design scenarios. The number of batteries (equivalently the battery storage capacity) and the feed pressure can be included as design parameters, depending on the selected scenario. The bounds of *N_v_* and *N_WT_* were chosen based on a prior grid search. The lower bound on *N_b_* is arbitrary, while the upper bound is evaluated based on the maximum storage capacity of 5 × 10^9^ kWh. The latter is determined based on prior simulation of the system. The lower bound of *P_f_* was chosen, such that it exceeds the osmotic pressure and the pressure drop across the unit. The maximum value of *P_f_* was chosen based on the maximum reported value in the literature. The nonlinear algebraic equations (*f*) represent the RO model equations. As mentioned earlier and shown in Figure 4, the design procedure includes solving the RO model in the inner loop to compute *WC*, *LPRH*, and *LPRA*.

We will use the terminology of variable feed pressure when dealing with feed pressure being altered by the RO model loop, and fixed feed pressure when it is altered by the design loop. For clarity, we will denote the outer loop as the design loop, and the inner loop as the RO loop. Moreover, when the design variable is denoted as “fixed” it means it has a constant value over the entire year, while the term “variable” means it changes each hour over the whole year. The “fixed” value of the design parameter is determined by the optimization (design loop).

## 5. Results and Discussion

Designing a rigid RO system, i.e., fixed number of turbines, number of vessels, a fixed number of batteries, or feed pressure while the wind power is alternating cannot provide the optimum solution for all operating conditions. This is because the alteration in wind speed is very random. Therefore, a better way to deal with this situation is to allow some of the design parameters to be variable to adapt to the randomness in the wind source, i.e., to vary on an hourly basis. Adaptation of the turbines and/or batteries is not recommended, because they are expensive, and their adaptation is more complicated practically. Hence adaptation of these parameters is excluded here. Thereby, the feed pressure and the number of active vessels will be adapted. Adaptation of feed pressure may provide good performance. However, because the number of the active vessels is fixed, the supplied power per vessel will vary, and so will the feed pressure and flow rate. In this case, there will be occasions of very low *P_wv_* and hence low production rate, or very high *P_wv_* and consequently saturation of the pump, leading to a portion of applied energy being wasted. On the other hand, fixing *P_wv_* will lead to a steady and smooth operation of the RO vessels, leading to a consistent production rate. In due course, the number of active vessels will vary hourly to absorb the wind energy variations.

The results of the optimization to attain the desired yearly production are listed in Table 2. In this table, various cases for the objective function and options of the design variables are considered for comparison and analysis. Two distinct objective functions were considered in the optimization problem, i.e., *LPA* and *WC*. Within the first objective function, two forms were considered, i.e., raw value (*LPA*) and absolute value (abs*LPA*). Within the second objective function, three forms were chosen, the sole objective function (*WC*), objective function subject to inequality constraints (*WCLPA*), and objective function subject to inequality constraint (*WCLPA*eq). For *WCLPA*, the inequality constraint is *LPRA* ≤0, and for *WCLPA*eq, the equality constraint is *LPRA* = 0. Under each case of the above cases, two options were tested, either fixed *P_wv_* or fixed *N_v_*. When *P_wv_* is fixed, *N_v_* will vary hourly and vice versa. This is because the two variables are interrelated via *P_wv_* = *P_wt_*/*N_v_*. When *N_v_* is fixed, all vessels will receive the same fluctuating power, while when *P_wv_* is fixed, only the active set of vessels will receive the same constant value of power. Note that a variable *N_v_* implies that a certain set of the total available vessels will be active. This can be achieved using an automatic control that enables and disables certain sets of vessels. Finally, Table 2 includes the results for the case of fixed RO pressure (determined by the outer loop) and variable pressure (determined by the inner loop).

**Figure 6:**
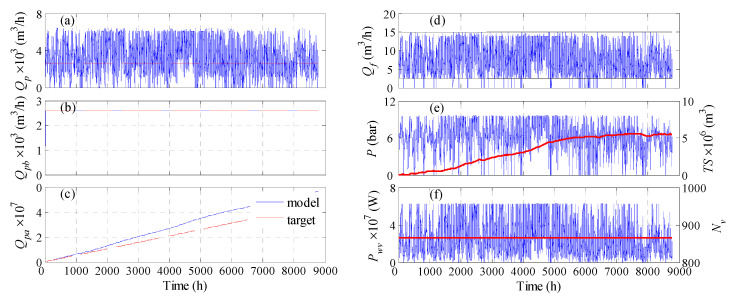
Optimization results for the case of variable pressure and optimized *N_WT_* and *N_v_* for satisfying the annual production rate using storage tanks; *Q_p_*: hourly production rate, *Q_pb_* hourly water supply in the pipeline, *Q_pa_*: accumulative production, *Q_f_*: hourly fee flow rate, *P*: feed pressure; *P_wv_*: supplied power per vessel.

For the fixed pressure case, three design variables were considered: *N_WT_*, *P*, and (*P_wv_* or *N_v_*). In general, the optimization results based on cost minimization (*WC*, *WCLPA*, and *WCLPA*eq) managed to reduce the water cost substantially compared to the other objective functions (*LPA*, abs*LPA*), by obviously reducing the number of process units, i.e., vessels and turbines. The water cost can be as low as 0.53 $/m^3^. However, this situation sacrifices the production rate as indicated by large values for *LPRA* and *LPRH*. In fact, the annual deficit can be as high as 73%. This is because decreasing the number of units, especially *N_WT_*, will lessen the available power and hence the production rate. Even when the cost objective function is augmented with equality and inequality constraints, to maintain a low loss of production, the optimization results in suboptimal solutions. This is because the cost and production are competing functions. However, since the main goal is to achieve the desired water demand, these results are not acceptable. On the other hand, when the loss of production probability is minimized (*LPA*, abs*LPA*) full satisfaction of the annual production is obtained as indicated by the negative values, i.e., surplus of water. However, this was attained at the expense of a larger number of units, and consequently, a higher water cost. The water cost can be as high as 18.5 $/m^3^, especially when the raw *LPRA* is minimized. Since the goal is to achieve the desired water production rather than producing excess water, we tried optimizing the absolute value of *LPRA*. In this case, the minimum possible value of *LPRA* is zero. It is obvious that a lower number of units is obtained, leading to much lower cost for water production, which falls in the range of 3.4–4.2 $/m^3^. In this case, the annual loss of production ratio changed from surplus to null or a very minor deficit (0.2%). This also affected the hourly loss of production, which increased from 0.2% to 2.1%. Accordingly, we can consider the result that correspond to minimizing raw *LPA* to be the best, despite its higher water cost, because it provides the best water production, with *LPRHb* as low as 0.2%. Obviously, zero *LPRHb* was not achieved because the early production losses around the first 100 h cannot be compensated for by the stored water in the storage tanks. At these early stages, the storage tanks were not sufficiently filled, as shown in Figure 6e. Figure 6a illustrates the actual production rate, while Figure 6b shows the water production after redistribution, i.e., storing the excess water and compensating for the losses. The latter demonstrates how unresolved water deficit occurs in the first 10 h of operation. Figure 6c depicts the accumulated produced water, and compares it to that of the target, which shows a clear surplus, as numerically manifested by the negative value for *LPRA*. Figure 6d–f show how the feed flow rate, feed pressure, and power per vessels vary with time due to variations in the wind speed. Some zero values were reported for these variables because the corresponding wind power was zero at these instants. The maximum amount of stored water is given in Table 2, which indicates higher amounts are stored for the *LPA* and *absLPA* cases compared to the other cases, which is intuitive. Note that a large number of storage tanks is required due to the large plant capacity. Regarding the options of using *P_wv_* or *N_v_* as the design parameter, minor effects can be observed. Notably, lower water cost is obtained when *N_v_* is used as the design variable because it incurs a smaller number of vessels. This is true for all optimization cases. In due course, fluctuating *P_wv_* is generated, which can be considered as detrimental to the pumps and membrane, as the operation (pressure and flow rate) is not smooth. Nevertheless, other researchers considered that turbulent operation of the RO unit is favorable, as it attenuates the adverse effect of concentration polarization [35]. As far as using *P_wv_* as the design parameter, different active sets of vessels will be triggered every hour but will be accompanied by a smooth operation, because the supplied power per vessel will be constant over the entire year. However, the water cost is adversely influenced because the capital cost is based on the maximum active vessels, not the average. This result agrees with the results published by Goosen et al. [41], which specified that the economic viability of variable operation is dependent on the extent to which it affects membrane performance and lifetime. Moreover, the optimization may necessitate large values for *P_wv_*, which may reach up to 13,792.5 W. This will lead to triggering very low, or even null, RO vessels, which causes improper utilization of the available wind power. The simulation was set such that no RO vessels are deployed if the available wind power is less than the design value of *P_wv_*.

Table 2 also illustrates the optimization results when a variable feed pressure is utilized. In this case, only two design variables are involved, namely *N_WT_* and *N_v_* or *P_wv_*. In general, the same previous trends were observed when comparing the optimization of *LPA* generations with that of the *WC* generations. Specifically, *LPA* generations provide better satisfaction of the water production, but at higher water cost. Moreover, the same previous trends were also observed when comparing *P_wv_* and *N_v_* as a design variable. The notable difference is that the water cost for an *LPA* case using variable pressure is much less than that for the same case with fixed feed pressure. The explanation of this behavior is that allowing the feed pressure to vary with wind fluctuations led to better utilization of the available wind power. In the fixed *N_v_* case, feed pressure varied with varying *P_wv_* incurring a lesser number of wind turbines. While in the fixed *P_wv_* case, the variable feed pressure permits the use of larger *P_wv_*, and hence a lower number of vessels. In both cases, the capital cost, and subsequently the water cost, is affected remarkably. Although, the annual production decreased compared to the fixed pressure case, the hourly production losses ratio remained as good as before. Note that the minimum *LPRHb* cannot be exceeded, for the same reason as mentioned earlier. Interestingly, the reduction in *LPRA* helped to minimize the required water storage capacity, which in turn influenced the capital cost positively. The variable feed pressure may have another advantage over the fixed operation, especially during realistic implementation. For example, when a disturbance occurs during operation, such as a sudden change in the feed salinity or membrane fouling, the automatic control may adjust the feed pressure to maintain the required production and water quality. In due course, the *LPA* strategy using variable pressure with *N_v_* as the design parameter is considered the best choice. The results confirms the analysis performed by Park et al. [11], which concluded that if short-term energy storage or buffering is to be considered, as it reduces the requirement of having the system running constantly, then the emphasis is shifted to the amount of power available to restart the system rather than the length of down-time.

**Figure 7:**
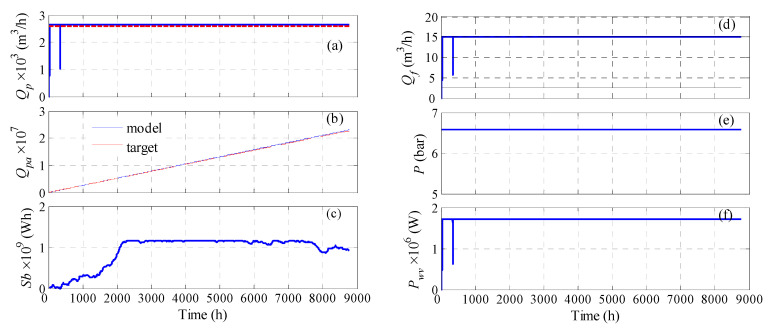
Optimization results for the case of fixed pressure and optimized *N_WT_*, *N_v_*, and *N_b_* for satisfying the hourly production rate via energy storage; *Q_p_*: hourly production rate, *Q_pa_*: accumulative production, *Sb*: Battery storage capacity; *Q_f_*: hourly fee flow rate, *P*: feed pressure; *P_wv_*: supplied power per vessel.

Next, we examine the case when the optimization problem is solved such that it satisfies the hourly water demand. In due course, a battery bank for energy storage is used instead of water storage tanks. The results are given in Table 3. Three situations of the optimization problem were tested. One considered minimizing the *LPRH*, the second *WC*, and the third *WC* augmented with equality constraints on *LPRH*. For each case, two options were attempted, i.e., fixed *P_wv_* or fixed *N_v_*. For the cases where fixed feed pressure was sought, four design parameters were utilized, namely *N_WT_*, *P*, *Sb*, and *P_wv_* or *N_v_*. As can be seen, when the water cost is minimized, with or without constraints, low water cost can be obtained. However, this is at the expense of loss of hourly production, as the system will suffer from an 11 to 22% deficit in hourly production. Even when constraints on *LPRH* are incorporated, the optimization yields a slight improvement in water production when *P_WV_* is used as a design parameter, and a slight degradation when *N_v_* is used as the design parameter. Of course, this was accompanied by a slight increase in the water cost, as it incurred an increase in the processing units, as well as the energy storage capacity. In both cases (*WC* and *WCLPH*) the reduction of water cost was achieved mainly by lowering the battery storage capacity, and secondarily, by lowering the process units in some cases. On the other hand, when the optimization problem focuses on minimizing *LPRH*, better hourly production can be obtained compared to the cases of *WC* and *WCLPH*. Specifically, the hourly production loss is lowered to 0.2–1.6%. This was achieved by substantially increasing the capacity of the energy storage device, which led to an expensive water cost of 19.7–29.9 $/m^3^. Unlike the design based on the annual production, using *P_wv_* as a design parameter outperformed *N_v_*. Specifically, the *P_wv_*-based design provided a lower water cost and less hourly water deficit. Note that the *N_v_*-based design has less power surplus than that of *P_wv_*-based design, hence further improvement of *LPRH* by energy transportation is not possible. Moreover, the *P_wv_*-based design still has an energy surplus (negative *LPSb*), but it is useless because it accumulates after more than 500 h of operation. Accordingly, a minimum hourly water deficit of 0.2% still exists which cannot be exceeded. We can see that 100% satisfaction of *LPRH* is not possible because there is a shortage in the production up to the first 500 h (Figure 7a). Note that increasing the generated power during that period by increasing *N_WT_* will not help because there are instances of zero *Q_f_* (Figure 7b), as the corresponding wind power/speed is zero. Moreover, during this initial period of operation, the batteries are insufficiently charged (Figure 7c) to compensate for the power loss. Figure 7f illustrates how the optimization managed to keep *P_wv_* as close to the required load as possible, except for the initial 1500 h, where insufficient wind power was supplied accompanied by insufficiently charged batteries. This in turn causes an unstable feed flow rate, as shown in Figure 7d, and consequently an unstable production rate (Figure 7a). Hence, the maximum achievable *LPRH* is limited by this situation, which can be resolved by recharging the batteries. However, as far as the simulation is concerned, this limitation should be considered when comparing the different scenarios. Nevertheless, this situation could be eliminated when operating continuously, whereby the battery will be sufficiently charged in the following year.

Table 3 also lists the results when the feed pressure was allowed to vary on an hourly basis in the RO loop during the overall optimization solution. As usual, the cost minimization strategy provided low water cost but unacceptable water production, both annually and hourly. Incorporation of constraints on the hourly production rate improved the losses in the hourly production ratio to 17–18%, compared to 26.7–28.3% for the case without constraining the production rate. These results are associated with a slight increase in the water cost to 1.36–1.87 $/m^3^, compared to 0.5–0.96 $/m^3^ for the unconstrained production. Nevertheless, these outcomes are considered unacceptable since water production is not accomplished. Considering the minimization of *LPRH*, interesting results were obtained. The *N_v_*-based design was outperformed by that of *P_wv_*, in terms of a slightly lesser water cost. However, the *N_v_*-based design was inferior to that of *P_wv_*, in the sense of percentage losses on the hourly production rate. The higher cost is due to acquiring a lower number of turbines and vessels. The *P_wv_*-based design provided a high water cost but with marginally lower hourly production losses of 0.6%. Comparing the optimal results of the variable pressure and fixed pressure for the *LPH* scenario, the variable pressure is inferior to constant pressure in terms of supplying the desired hourly production (i.e., minimum *LPH*) and lower water cost. In due course, the *LPH* strategy with fixed feed pressure using *P_wv_* as the design parameter is taken as the best choice.

**Figure 8:**
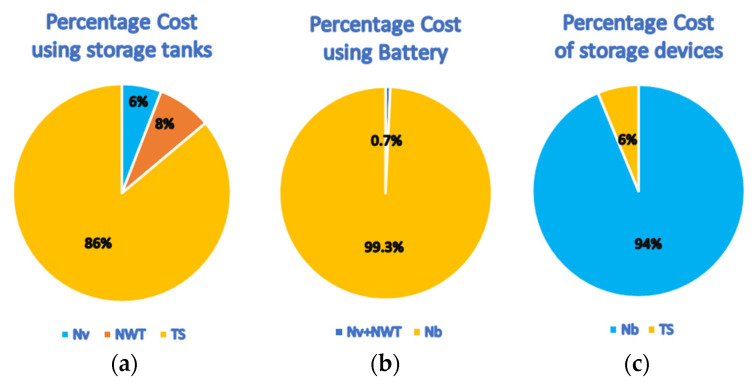
Contribution of the major plant components in the capital cost.

In conclusion, the outcomes of the annual-based strategy (Table 2) differ from those of the hourly-based strategy (Table 3) in two issues. First, using variable pressure is preferable over fixed pressure, and using *N_v_* as a design parameter is desirable over *P_wv_*. Moreover, the *LPA* strategy provided less expensive water costs at the same percentage of hourly production losses. The water cost in the *LPA* mode is 7.42 $/m^3^, while it is 18.67 $/m^3^ in the *LPH* mode, which corresponds to more than 50% savings. Generally, designing the process based on hourly production is more challenging because it is based on the desired energy load instead of the desired production. The desired production is certain, but the desired load is not decisive, as will be explained in the following. Numerically, the satisfaction of the desired production is straightforward but complicated for the desired energy load. The desired load is computed based on specific operating conditions for the feed pressure (corresponding flow rate) and the number of vessels that provide a certain production rate. This means there are several combinations of the operating conditions that provide the same desired production but at different loads. Therefore, when allowing pressure and number vessels to vary, it becomes very difficult to attain the desired production rate and the designated load simultaneously. Furthermore, the design strategy based on hourly production is more costly, due to the large cost of the battery and its associated inverters. Figure 8a depicts the contribution of the major components of the plant to the capital cost for the annual strategy using storage tanks. The storage tank consumes the largest portion, with 86% of the total. Figure 8b shows the proportion of the component cost in the case of hourly strategy using batteries. The cost of energy storage systems dominates the total cost by 99.3%. Finally, Figure 8c compares the fixed investment of the storage tank with that of the energy storage system, which reveals how the energy storage devices are more expensive compared to the conventional storage tanks. Note that the comparison of these elements is based on the best cases provided by Table 2 and Table 3.

**Figure 9:**
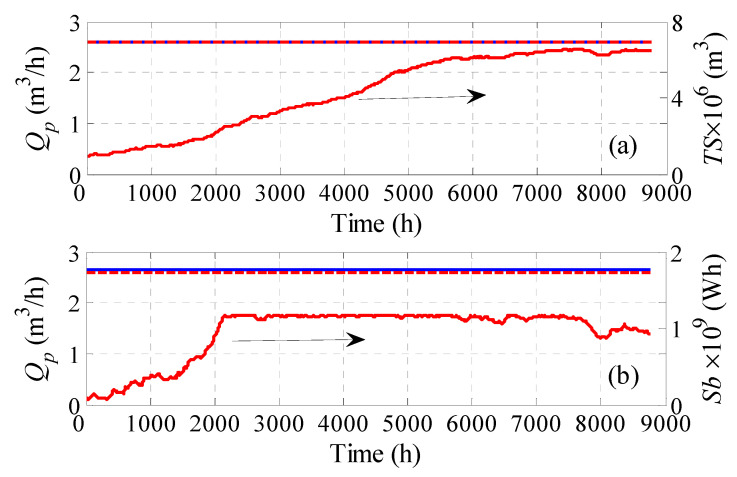
Plant production rate when the plant storage is initialized, (**a**) using storag tanks; (**b**) using energy storage; *Q_p_*: hourly production rate, *Ts*: Tank storage capacity; *Sb*: Battery storage capacity.

The best design results shown in Table 2 and Table 3 exhibit minor losses in their hourly production. As mentioned earlier, this is attributed to the fact that the tank and battery storage were initialized with a zero value. Hence, we re-simulated these cases using the obtained optimal values for the design parameter, but with the storage devices being initialized with a nonzero value. Specifically, the storage tank was filled with 1 × 10^6^ m^3^ of water, and the battery was charged with 1 × 10^8^ Wh of energy. The outcome of the simulation is depicted in Figure 9. It is obvious that the hourly water production is perfectly fulfilled for both cases. Note that these results were obtained by simulating the plant without optimization.

According to the results shown in Table 2 and Table 4, variable feed pressure may lead to the best design outcome for the annual-based scenario. Conversely, for the hourly-based scenario, the use of variable pressure still provides reasonable outcomes, but not as good as that of the fixed feed pressure. Nevertheless, the use of variable feed pressure via automatic control may bestow additional operational benefits. For example, it can reject the effect of internal and external disturbances during daily operation. To demonstrates this behavior, we simulated the plant under the existence of disturbances. Specifically, the disturbances comprised a sudden increase in the feed salinity from 1.0 to 1.5 kg/m^3^ and a sudden drop in the membrane permeability by 10%. The latter resembles the influence of membrane fouling. The plant will be simulated using the optimal conditions found in Table 2 and Table 3. Specifically, we selected the case of annual production with variable feed pressure and *N_v_* as a design parameter, and denoted it as VPA_*N_v_*. In addition, we chose the case of hourly production with fixed feed pressure and *P_wv_* as the design parameter, and denoted it as FPH_*P_wv_*. The comparison results are shown in Table 4. For both cases, the storage devices were pre-initialized properly, as in Figure 9. Clearly, for FPH_*P_wv_*, the plant performance deteriorates as the permeate purity increases to 0.62 kg/m^3^, and the loss in hourly production rises to 2.5%. This is because the feed pressure is kept constant while the process is suffering from disturbances. It should be noted that RO is a pressure driven separation process, where the transmembrane pressure plays an important role in its operation. On the other hand, VPA_*N_v_* managed to overcome the impact of the disturbances by increasing the feed pressure. Note that the pressure is variable each hour here, but the average value is given as an indicator. In this case, the loss of hourly production is kept as low as zero, but the water purity increased to 0.3 kg/m^3^, which is still less than the target value of 0.5 kg/m^3^.

## 6. Conclusions

This work was concerned with an investigation of ways to optimize the design and operation of a wind-driven RO desalination plant. The plant has a large capacity of 2592 m^3^/h, and requires a large steady load of 1780 kW each hour. Designing and operating the plant under heavily fluctuations in wind power is a challenging task. Different design and operation scenarios were optimized to find the best operation strategy, and the best values for the design parameters that make the plant produce the required water demand. The following are the obtained observations:

Optimizing the plant to minimize the water cost does not necessarily lead to the desired operation of maintaining the required water demand. For example, the lowest water cost of 0.53 $/m^3^ using water storage is associated with 43% losses in the annual production. Similarly, the lowest water cost of 0.4 $/m^3^ using energy storage is associated with a 10.3% deficit in the hourly production.

Optimizing the plant to satisfy the required production rate using storage tanks is preferable over utilizing energy storage systems, because it leads to less expensive water costs, and is less complicated and more easy to operate. For the same minimum losses of 0.2% in the hourly production, the water cost for the water-storage approach is 7.42 $/m^3^, compared to 19.67 $/m^3^ for the battery-storage approach.

Operating the RO vessels under variable pressure provides better attenuation of the wind intermittency and disturbances. Considering the *LPH* case, *LPS* is reduced from −3.33 to −0.13 when fixed feed pressure is used, which corresponds to 60% utilization of the surplus power. However, for the variable feed pressure case, *LPS* is reduced from −0.14 to 0.121, which corresponds to 100% utilization of the surplus power.

- Operating the plant under fixed power per vessel and fixed RO pressure leads to a smoother operation and production rate.
- The water cost can be 7.42 $/m^3^ when storage tanks are used, and 19.67 $/m^3^ when batteries are used, that guarantee zero loss of hourly production probability.

The optimal design was obtained for certain operating conditions. In a real application, these conditions may change, and disturbances may occur, such as membrane fouling and/or failure of some units, as reported by Ben et al. [42] and Mito et al. [20]; which leads to the conclusion that it is difficult to determine the optimal configuration with classical techniques. Further investigation of how to manipulate the feed pressure and/or a number of active vessels online to improve the overall performance of the plant will be addressed in future work.

## Figures and Tables

**Table 1:** Geometric Specification of the Membrane Module [35].

Parameter	Value
The hydraulic diameter of channel, *d_h_* (mm)	0.78045
Height of spacer channel, *h_sp_* (mm)	0.593
Void fraction of the spacer, *ε* (porosity)	0.9
Length of membrane, *L* (m)	1
Width of membrane, *W* (m)	37
Active area of the membrane, *A_e_* (m^2^)	37
Reference water permeability, *A*_0_ (m^3^/(h.bar))	19.43 × 10^−4^
Reference solute permeability, *B*_0_ (m^3^/h)	78.55 × 10^−5^

**Table 2:** Optimization results for satisfying the yearly production.

Case		*LPA*		abs*LPA*		*WC*		*WCLPA*		*WCLPA*eq	
		ϕ = *LPRA*		ϕ = |*LPRA*|		ϕ = *WC*		ϕ = *WC*, *C_ineq_* = *LPRA*		ϕ = *WC*, *C_eq_* = *LPRA*	
Option		Fixed *P_wv_*	Fixed *N_v_*	Fixed *P_wv_*	Fixed *N_v_*	Fixed *P_wv_*	Fixed *N_v_*	Fixed *P_wv_*	Fixed *N_v_*	Fixed *P_wv_*	Fixed *N_v_*
Fixed Pressure	*N_v_* (max)	(3462)	701	(1535)	668	(427)	418	(232)	654	(412)	652
	*N_WT_*	100	100	81	84	60	55	53	59	69	58
	*P_wv_*, *W*	1732.3	Vary	3166.0	Vary	8496.8	Vary	13,792.5	Vary	10,032.4	Vary
	*P*, bar	5.0	5.3	8.1	7.5	12.2	8.9	15.8	9.8	8.9	11.2
	*LPS*	−0.4	−0.396	−0.13	−0.183	0.15	0.225	0.26	0.176	0.04	0.185
	*LPRA*	−1.007	−0.378	0	0.002	0.504	0.434	0.731	0.454	0.521	0.543
	*LPRH*	0.122	0.147	0.29	0.279	0.528	0.469	0.731	0.517	0.54	0.576
	*LPRHb*	0.002	0.0019	0.021	0.0165	0.504	0.4339	0.731	0.454	0.521	0.5432
	*WC*, $/m^3^	18.46	10.14	4.24	3.41	0.76	0.53	0.86	0.75	0.79	0.71
	*TS*, m^3^	2.3 × 10^7^	8.7 × 10^6^	2.2 × 10^6^	1.9 × 10^6^	1.0 × 10^4^	1.5 × 10^4^	0	5.6 × 10^4^	8.3 × 10^3^	1.4 × 10^4^
Variable Pressure	*N_v_* (max)	(1202)	866	(1024)	800	(153)	1000	(234)	1006	(261)	1006
	*N_WT_*	100	90	100	71	77	60	50	50	50	50
	*P_wv_*, *W*	4989.3	Vary	5854.5	Vary	30331.8	Vary	12,918.9	vary	11,541.9	Vary
	*LPSP*	−0.35	−0.21	−0.35	0.04	−0.04	0.19	0.32	0.33	0.32	0.32
	*LPRA*	−0.257	−0.241	−0.077	−0.037	0.716	0.008	0.585	0.127	0.568	0.125
	*LPRH*	0.224	0.153	0.268	0.212	0.716	0.24	0.588	0.289	0.575	0.288
	*LPRHb*	0.003	0.002	0.01	0.009	0.716	0.016	0.585	0.127	0.568	0.125
	*WC*, $/m^3^	7.9	7.42	5.04	3.6	0.75	3.14	0.58	1.39	0.6	1.39
	*TS*, m^3^	6.3 × 10^5^	5.6 × 10^6^	3.3 × 10^5^	3.3 × 10^7^	0	1.7 × 10^6^	133.4	4.9 × 10^5^	313.0	4.91 × 10^5^

**Table 3:** Optimization results for satisfying the hourly production; *WC*: water cost, *LPRH*: hourly loss of power probability.

Case		*LPH*		*WC*		*WCLPH*	
		ϕ = *LPRH*		ϕ = *WC*		ϕ = *WC*, *C_eq_* = *LPRH*	
Option		Fixed *P_wv_*	Fixed *N_v_*	Fixed *P_wv_*	Fixed *N_v_*	Fixed *P_wv_*	Fixed *N_v_*
Fixed pressure	*N_v_* (max)	(352)	665	(738)	610	(246)	667
	*N_WT_*	95	78	79	97	88	70
	*P_wv_*_,_ *W*	4888.5	vary	2317.1	vary	6955.3	vary
	*P*, bar	6.6	7.17	3.4	5.02	11.3	5.82
	*S_b_*, Wh	1.16 × 10^9^	1.94 × 10^9^	1.12 × 10^6^	1.11 × 10^6^	5.28 × 10^6^	1.35 × 10^7^
	*LPS*	−0.33	−0.09	−0.11	−0.361	−0.24	0.02
	*LPSb*	−0.13	0.16	−0.08	−0.333	−0.15	0.143
	*LPRA*	−0.016	0.016	−0.418	−0.168	0.457	0
	*LPRH*	0.002	0.016	0.103	0.124	0.457	0.161
	*WC*, $/m^3^	19.67	29.91	0.4	0.42	0.67	0.65
Variable pressure	*N_v_* (max)	(954)	551	(1289)	807	(1907)	666
	*N_WT_*	81	76	75	54	70	60
	*P_wv_*_,_ W	1806.9	vary	3507.9	vary	2195.5	vary
	*S_b_*_,_ Wh	2.6 × 10^9^	3.0 × 10^9^	4.0 × 10^6^	1.1 × 10^6^	5.0 × 10^7^	5.0 × 10^7^
	*LPS*	−0.124	−0.066	−0.056	0.241	0.021	0.16
	*LPSb*	0.121	0.188	0.012	0.263	0.187	0.29
	*LPRA*	−0.24	−0.01	0.239	0.187	0.089	0.088
	*LPRH*	0.006	0.014	0.283	0.267	0.189	0.171
	*WC*, $/m^3^	27.45	25.94	0.96	0.52	1.87	1.36

**Table 4:** Comparing operation under disturbances.

Case	VPA-*N_v_*		FPH-*P_wv_*	
	Nominal	Disturbed	Nominal	Disturbed
*N_v_*	866	866	352	352
*N_WT_*	90	90	95	95
*P_av_*	6.36	7.26	6.6	6.6
*LPSP*	−0.214	−0.214	−0.335	−0.335
*LPRPA*	−0.24	−0.066	−0.016	0.025
*LPRPH*	0.153	0.218	0.002	0.025
*LPRHb*	0	0	0	0.025
*Cp_av_*	0.26	0.30	0.50	0.62

## Data Availability

The data presented in this study are available on request from the corresponding author.

## Appendix A

### Appendix A.1. Algorithm S1: Variable Feed Pressure

The numerical solution of the RO model with variable pressure (specified by the RO system) can be described by the following algorithm (S1):

1. Given the process parameters and operating conditions such as *C_f_*, *P_w_*, *N_v_*, and $R_{c}^{\prime }$.
2. Set $P_{wv}=P_{w}/N_{v}$
3. Solve the following optimization problem:
  (A1) $$\underset{P_{f},C_{p}^{o},C_{m}^{o}}{\min}\varphi ={\left(R_{c}-R_{c}^{\prime }\right)}^{2}$$

Subject to:

(A2) $$C_{p}\leq C_{pd}$$

(A3) $$C_{p}-C_{p}^{o}=0$$

(A4) $$C_{m}-C_{m}^{o}=0$$

(A5) $$3<P_{f}\leq 40$$

(A6) $$Q_{fmin}\leq Q_{fv}\leq Q_{fmax}$$

The last constraints (A5), (A6) are imposed to ensure the obtained result respects the safe operation window. Note that *R_c_*, *C_m_*, *C_p_*, and *Q_fv_* are obtained by solving the RO model equations.

### Appendix A.2. Algorithm S2: Fixed Feed Pressure

The numerical solution of the RO model using fixed feed pressures (specified by the design loop) can be explained by the following algorithm (S2):

4. Define all process parameters and operating conditions such as *C_f_*, *P_w_*, *P_f_*, *N_v_*.
5. Set $P_{wv}=P_{w}/N_{v}$
6. Compute Q_fv_ using Equation (5)
7. Assume initial values for $C_{p}=C_{p}^{o}\;\&\;C_{m}=C_{m}^{o}$
8. Compute Equations (7)–(19)
9. If $C_{p}-C_{p}^{o}<\varepsilon \;\&\;C_{m}-C_{m}^{0}<\varepsilon$, where *C_m_* and *C_p_* are computed from Equations (18) and (19), respectively, proceed to step 7, otherwise set $C_{p}^{o}=C_{p}\;\&\;C_{m}^{o}=C_{m}$ go back to step 5
10. Compute *Q_w_* and *R_c_*, Stop.

## Appendix B

### Economic Analysis

**Table A1:** Fixed Capital Investment.

Component	Correlation
Capital cost for intake pumping and pre-treatment, *CC_IP_*	*CCIP* = 996(Q_f_)^0.8^ [43]
Capital cost for the high pressure pumps, *CC_HP_*	$\log_{10}C_{hp}=3.3892+0.0536\log_{10}W_{HP}+0.1538{\left(\log_{10}W_{HP}\right)}^{2}$ [43]
	*CC_HP_* = *N_HP_* × *C_hp_* [43]
Capital cost of wind turbine. *CC_WT_*	*CC_WT_* = *N_WT_* × *P_WT_* × *C_WT_* [44]
Capital cost for membranes, *CC_M_*_b_	*CC_MB_ = f_MB_ C_MB_ N_MB_* [30]
Capital cost of pressure vessels, *CC_PV_*	*CC_PV_ = f_PV_ C_PV_ N_PV_* [30]
Capital cost of pipes, *CC_pipes_*	*CC_pip_*_e_ = 0.4*C_E_* [45]
Capital cost of water storage tanks, *CC_st_*	*CCst* = *TS^m^* × *Cst*
Capital cost of battery bank, *CC_bt_*	*CC_bt_* = *Sb^m^* × *C_bt_*
Capital cost of inverters, *CC_inv_*	*CC_inv_* = *Sb^m^* × *C_inv_*
Capital cost: *CC*	*CC* = *CC_PV_* + *CC_MB_* + *CC_pipe_* + *CC_HP_* + *CC_IP_* +*CC_WT_* + *CC_st_* + *CC_inv_* + *CC_bt_* [30]
Cost of site development, *CC_site_*	*CC_site_* = 0.1*CC* [30]
Direct capital cost, *DCC*	*DCC* = *CC + CC_site_*
Indirect capital cost, *ICC*	*ICC* = 0.27*DCC* [30]
Total capital cost, *TCC*	*TCC = DCC + ICC*
Annualized capital cost, *ACC*	$ACC\left(\frac{\$}{y}\right)=TCC\left(\$\right)\frac{i{\left(i+1\right)}^{n}}{{\left(i+1\right)}^{n}-1}$ [30,46]

**Table A2:** Annual Operating Cost.

Item	Component	Correlation
*OC_O&M_*	Annual labor cost	0.01$/m^3^ [47]
	Annual maintenance cost of potable water	0.01 $/m^3^ [47]
	Annual chemical cost of potable water	0.04 $/m^3^ [47]
	Annual insurance cost	0.05 × *TCC* [39]
*OC_Mb_*	Annual renewal cost for membrane	*C_MB_ × N_MB_/n_LT_* [30]
*OC_WT_*	Annual maintenance cost for turbines	*C_Mnt-WT_ × N_WT_ × P_WT_* [44]
*OC_st_*	Annual maintenance cost of storage tanks	*TS^m^ × C_stmt_*
*OC_bt_*	Annual renewal cost for battery	*C_bt_ × Sb^m^/n_bt_*
*OC_inv_*	Annual renewal cost for inverters	*C_inv_ × Sb^m^/n_inv_*
*OC_btmt_*	Annual maintenance cost for battery	*C_btmt_ × Sb^m^*
*TOC*	Total annual operating cost	*OC_power_ + OC_OM_ + OC_Mb_ + OC_WT_ + OC_bt_ + OC_inv_ + OC_btmt_*

**Table A3:** Operating and Design Parameters.

Item	Abbreviation	Value
Equipment corrective factor	*f*_MB_, *f*_PV_	1 [30]
Pump efficiency	*η_HP_*	0.6
Battery efficiency	*η_bat_*	0.85
Inverter efficiency	*η_inv_*	0.95
Pump operating power	*W_HP_*	55.56
Total pump power, kW	*W_HPt_*	27778
Required wind power, kW	*W_HP actual_*	1780
RO surface area, m^2^	*A*	37
Membrane unit cost, $/m^2^	*C_MB_*	10 [43]
Vessel unit cost, $	*C_PV_*	1000 [38]
Turbine unit cost, $/kW	*C_WT_*	1804 [44]
Turbine maintenance cost, $/y	*C_Mnt-WT_*	100 [44]
Water storage tank cost, $/m^3^	*C_st_*	110 [18]
Storage tank maintenance cost, $/(m^3^.y)	*C_stmt_*	2.5 [18]
Battery storage cost, $/kW	*C_bt_*	200 [27]
Inverter/converter cost, $/kW	*C_inv_*	700 [27]
Battery maintenance cost, $/kW	*C_btmt_*	25 [27]
Maximum battery storage capacity, Wh	*Sb^max^*	5 × 10^9^
Interest rate	*i*	0.08
Plant life, y	*n*	20
Membrane life time, y	$n_{LT}$	5
Wind turbine life time, y	$n_{WT}$	20
Battery life time, y	*n_bt_*	10 [27]
Inverter life time, y	*n_inv_*	10 [27]
Annual operating hours, h/y	$w_{hy}$	7884
